# Accuracy, repeatability, and reproducibility of T_1_ and T_2_ relaxation times measurement by 3D magnetic resonance fingerprinting with different dictionary resolutions

**DOI:** 10.1007/s00330-022-09244-x

**Published:** 2022-11-24

**Authors:** Krishna Pandu Wicaksono, Yasutaka Fushimi, Satoshi Nakajima, Akihiko Sakata, Sachi Okuchi, Takuya Hinoda, Sonoko Oshima, Sayo Otani, Hiroshi Tagawa, Yuta Urushibata, Yuji Nakamoto

**Affiliations:** 1 Department of Diagnostic Imaging and Nuclear Medicine, Graduate School of Medicine, Kyoto University, 54 Shogoin Kawahara-cho, Sakyo-ku, Kyoto, 606-8507 Japan; 2 Siemens Healthcare K.K., Shinagawa-ku, Tokyo, Japan

**Keywords:** 3D MRF, Dictionary resolution, Accuracy, Repeatability, Reproducibility

## Abstract

**Objectives:**

To assess the accuracy, repeatability, and reproducibility of T_1_ and T_2_ relaxation time measurements by three-dimensional magnetic resonance fingerprinting (3D MRF) using various dictionary resolutions.

**Methods:**

The ISMRM/NIST phantom was scanned daily for 10 days in two 3 T MR scanners using a 3D MRF sequence reconstructed using four dictionaries with varying step sizes and one dictionary with wider ranges. Thirty-nine healthy volunteers were enrolled: 20 subjects underwent whole-brain MRF scans in both scanners and the rest in one scanner. ROI/VOI analyses were performed on phantom and brain MRF maps. Accuracy, repeatability, and reproducibility metrics were calculated.

**Results:**

In the phantom study, all dictionaries showed high T_1_ linearity to the reference values (*R*^2^ > 0.99), repeatability (CV < 3%), and reproducibility (CV < 3%) with lower linearity (*R*^2^ > 0.98), repeatability (CV < 6%), and reproducibility (CV ≤ 4%) for T_2_ measurement. The volunteer study demonstrated high T_1_ reproducibility of within-subject CV (wCV) < 4% by all dictionaries with the same ranges, both in the brain parenchyma and CSF. Yet, reproducibility was moderate for T_2_ measurement (wCV < 8%). In CSF measurement, dictionaries with a smaller range showed a seemingly better reproducibility (T_1_, wCV 3%; T_2_, wCV 8%) than the much wider range dictionary (T_1_, wCV 5%; T_2_, wCV 13%). Truncated CSF relaxometry values were evident in smaller range dictionaries.

**Conclusions:**

The accuracy, repeatability, and reproducibility of 3D MRF across various dictionary resolutions were high for T_1_ and moderate for T_2_ measurements. A lower-resolution dictionary with a well-defined range may be adequate, thus significantly reducing the computational load.

**Key Points:**

*• A lower-resolution dictionary with a well-defined range may be sufficient for 3D MRF reconstruction.*

*• CSF relaxation times might be underestimated due to truncation by the upper dictionary range.*

*• Dictionary with a higher upper range might be advisable, especially for CSF evaluation and elderly subjects whose perivascular spaces are more prominent.*

**Supplementary Information:**

The online version contains supplementary material available at 10.1007/s00330-022-09244-x.

## Introduction

Magnetic resonance fingerprinting (MRF) is a new quantitative MRI technique that enables rapid, simultaneous estimation of multiple tissue properties, such as T_1_ and T_2_ values [1]. Since its inception in 2013, various potential clinical applications of MRF have been studied, including mesial temporal lobe epilepsy [2], brain tumors [3], Parkinson’s disease [4], frontotemporal lobe degeneration [5], and brain perfusion [6].

MRF applies pseudorandomized acquisition to create temporal incoherence, so that different tissues show unique signal evolutions, as the so-called fingerprint. These fingerprints are then matched to a predefined dictionary containing sets of predicted signal evolutions [7]. The dictionary thus becomes a crucial component of MRF reconstructions. A dictionary is determined by the number of anticipated tissues and combinations of system-related parameters. In general, dictionaries include T_1_ and T_2_ values [8]. Each parameter has ranges and step sizes, determining the dictionary resolution.

Three-dimensional (3D) MRF provides higher signal-to-noise ratio efficiency and spatial resolution compared to 2D MRF [9, 10]. Combined with a parallel imaging technique, such as generalized autocalibrating partial parallel acquisition (GRAPPA), 3D MRF can provide whole-brain imaging within a feasible time [11]. Although various MRF reproducibility studies have been performed [12–16], the implementation of dictionaries with differing ranges and step sizes on 3D MRF accuracy, repeatability, and reproducibility is not yet thoroughly investigated. This study investigated a 3D MRF reconstructed using various dictionary resolutions to evaluate measurement accuracy, repeatability, and reproducibility in the phantom and human brain.

## Materials and methods

This prospective study was performed in accordance with the Declaration of Helsinki and was approved by the local Ethics Committee. All volunteers provided written informed consent prior to enrollment.

### MRF acquisition and reconstruction

Two 3-T MRI units (MAGNETOM Prisma; Siemens Healthineers) with a 20-channel head coil were used for the phantom study, while a 64-channel head/neck coil was used in the human study. We implemented a commonly used 3D fast imaging with steady-state precession (FISP) MRF sequence, as described by Liao et al [11]. The acquisition details are as follows: in-plane resolution, 1 mm; field of view (FOV), 240 × 240 × 192 mm^3^; slice thickness, 2 mm; echo time (TE), 2.7 ms; repetition time (TR), 12–13 ms (varied with a Perlin noise pattern); flip angle (FA), 5–80° (varied sinusoidally); acceleration factor, 3; time points, 450 (420 for MRF, and 30 for the auto-calibration); scan time, 5.5 min.

Four dictionaries with equal ranges but different step sizes were used for pattern matching. The highest resolution dictionary, denoted as HRD, had 300 T_1_ entries (2:2:100 [minimum:step:maximum], 105:5:400, 410:10:1500, 1520:20:2500, 2550:50:3500, 3600:100:4500) and 300 T_2_ entries (1:1:150, 152:2:250, 255:5:400, 410:10:600, 620:20:1200, 1250:50:1900, 2000:100:2500). The moderately low resolution dictionary ( LRD-1) had 105 T_1_ entries (10:10:100, 120:20:1000, 1040:40:2000, 2100:100:4500) and 100 T_2_ entries (2:2:100, 105:5:150, 160:10:300, 350:50:1000, 1100:100:1700, 1900:200:2500). The subsequent low resolution dictionary (LRD-2) had 50 T_1_ entries (20:20:100, 140:40:1020, 1100:100:1900, 2100:200:4500), and 50 T_2_ entries (5:5:100, 110:10:160, 180:20:300, 400:100:1500, 1700:200:2500). Dictionary with the lowest resolution (LRD-3) had 25 T_1_ entries (40:40:240, 300:100:1200, 1200:200:2000, 2500:500:4500), and 25 T_2_ entries (10:10:100, 120:20:200, 250:50:400, 600:200:1000, 1500:500:2500). Additionally, we also performed experiment with a much wider range dictionary and finer step size, denoted as very high-resolution dictionary (VHRD), which had 1150 entries for both T_1_ and T_2_ (1:1:100, 102:2:1000, 1010:10:7000).

The dictionary generation is based on the extended-phase-graph (EPG) model, as described by Weigel [17]. For the estimation of the quantitative mappings, 3D volumes of multiple time points were normalized and pattern-matched voxel-wise to the corresponding dictionary using the maximum inner product method. MRF reconstruction was performed using a workstation with the following specification: CPU Intel Core i7 7800x 3.50 GHz; memory, 32 GB; GPU, Nvidia Geforce RTX 2080 Ti 11 GB.

### Phantom study

The International Society of Magnetic Resonance in Medicine/National Institute of Standards and Technology (ISMRM/NIST) phantom was scanned daily for 10 days in both scanners. This phantom consists of 14 spheres of each T_1_ and T_2_ array with specific values (T_1_, 23–1838 ms; T_2_, 5–646 ms). We determined to evaluate the measured values of phantom arrays number 1 to 7 (T_1_, 259–1838 ms; T_2_, 63–646 ms) as those arrays represent physiologic ranges of T_1_ and T_2_ values in the human brain. Reference T_1_ and T_2_ values of phantom arrays were obtained from the phantom manual provided by the manufacturer. We also evaluated the MRF measurement consistency in the boundary slices outside the phantom spheres. The phantom temperature before and after acquisitions was measured, and the average of the two was recorded.

### Volunteer study

Between October 2019 and April 2020, 39 healthy volunteers without any known neurological disease were enrolled. Twenty volunteers underwent MRF scans in both scanners, and the remaining underwent scans in either scanner. T_1_-weighted images were also obtained using a 3D magnetization-prepared rapid acquisition of gradient echo (3D-MPRAGE) sequence with the following parameters: TR, 1900 ms; TE, 2.6 ms; inversion time (TI), 900 ms; FA, 9°; FOV, 230 × 230 mm; matrix size, 256 × 256; slice thickness, 0.9 mm; and parallel imaging factor, 2.

### Post-imaging processing

A circular region of interest (ROI) 10 mm in diameter was drawn in the center slice of each T_1_ and T_2_ phantom array using ImageJ application (https://imagej.nih.gov/ij/). As the T_1_ arrays are located near the apex of the phantom and T_2_ arrays near the midline, the axial cross-section area for T_2_ arrays is larger than T_1_ arrays. Therefore, we opted to mask the phantom area with the same size for T_1_ and T_2_ arrays. In addition, two sets of 4 circular ROIs with the same diameter were drawn, each set in the upper and lower boundary slices outside the phantom spheres. Mean relaxation times were measured using the built-in function of ImageJ.

**Fig. 1:**
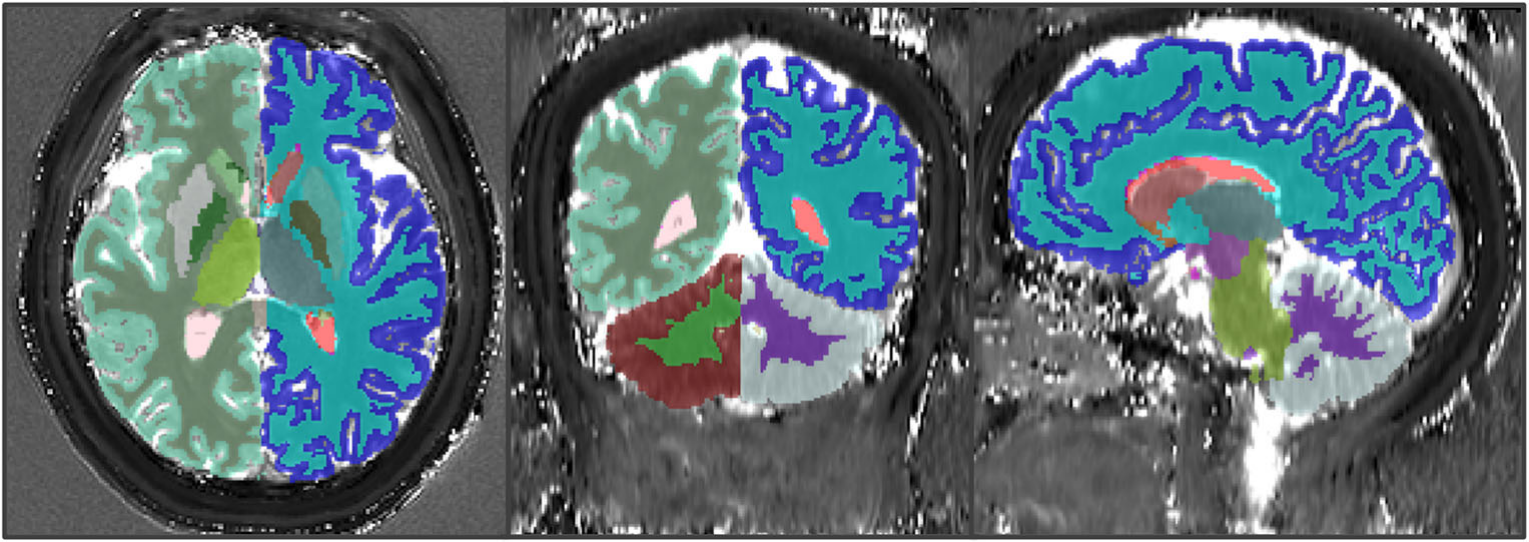
Example of T_1_ map overlaid with volumes of interest selected from FreeSurfer segmentation results

In the volunteer study, T_1_-weighted images were co-registered with MRF maps. Average normalized MRF maps were then created using the Diffeomorphic Anatomical Registration Through Exponentiated Lie Algebra (DARTEL) template in SPM 12 (https://www.fil.ion.ucl.ac.uk/spm/software/spm12/). T_1_-weighted images were segmented using FreeSurfer 7 (https://surfer.nmr.mgh.harvard.edu/), and several volumes of interest (VOIs) were chosen: cerebral white and grey matter, cerebellar white and grey matter, thalamus, caudate nucleus, putamen, globus pallidus, hippocampus, and lateral ventricle. Mean T_1_ and T_2_ values of those VOIs were extracted from each T_1_ and T_2_ map in the native space using the ITK-SNAP application (http://www.itksnap.org/) [18], as represented in Fig. 1.

### Statistical analysis

Statistical analyses were performed using commercially available software (MedCalc version 20.0; MedCalc Software Ltd.). Accuracy was assessed by linear regression and Bland-Altman (BA) relative difference plots between measured relaxation times (average from 10 days of measurements) and phantom reference values. The coefficient of variation (CV) over 10 days of scanning was calculated to evaluate repeatability. Reproducibility was determined by calculating BA plots, intraclass correlation coefficients (ICCs), and within-subject CVs between MRF maps from different scanners in phantom and healthy volunteers. Mean T_1_ and T_2_ values of brain VOIs between dictionaries were compared using ANOVA.

## Results

### Phantom study

**Fig. 2:**
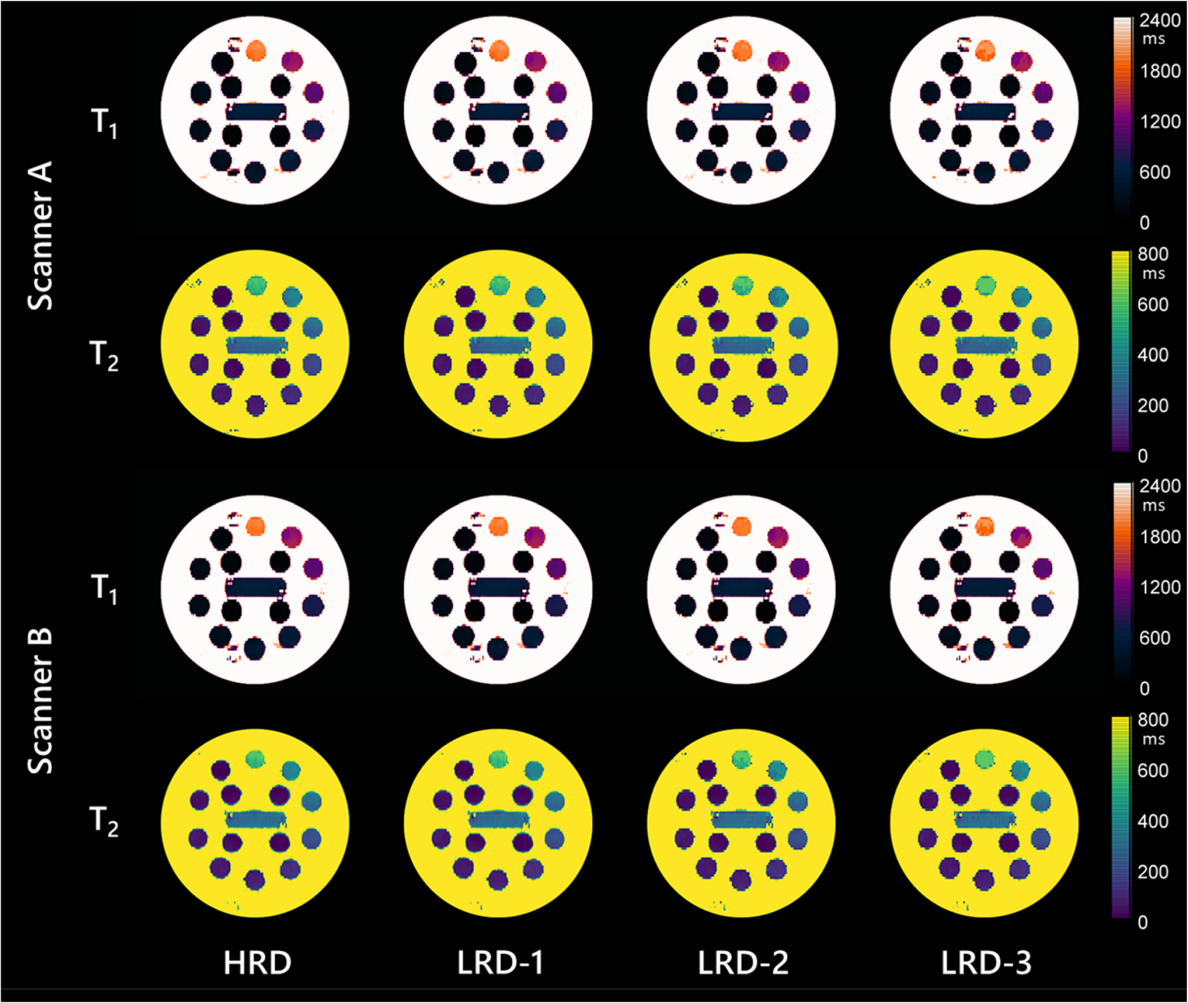
Representative T_1_ and T_2_ maps of the ISMRM-NIST phantom, reconstructed using the high-resolution dictionary (HRD; first column from left), moderately low-resolution dictionary (LRD-1; second column), low-resolution dictionary (LRD-2; third column) and very low-resolution dictionary (LRD-3; fourth column), scanned in two MR scanners. Window width was set at 2400 ms and window level at 1200 ms for T_1_ maps and at 800 ms and 400 ms for T_2_ maps. T_1_ and T_2_ maps of 4 dictionaries showed apparently similar results

Representative phantom images of T_1_ and T_2_ maps, reconstructed using each dictionary in each scanner, are shown in Fig. 2. Linearity between measured and reference values were excellent, with all dictionaries showing *R*^2^ > 0.99 and slope: 1.03 (HRD and LRD-1), 1.02 (LRD-2), 1.05 (LRD-3) for T_1_ arrays, and *R*^2^ > 0.98 and slope: 0.86 (HRD and LRD-1), 0.87 (LRD-2), 0.92 (LRD-3) for T_2_ arrays.

**Fig. 3:**
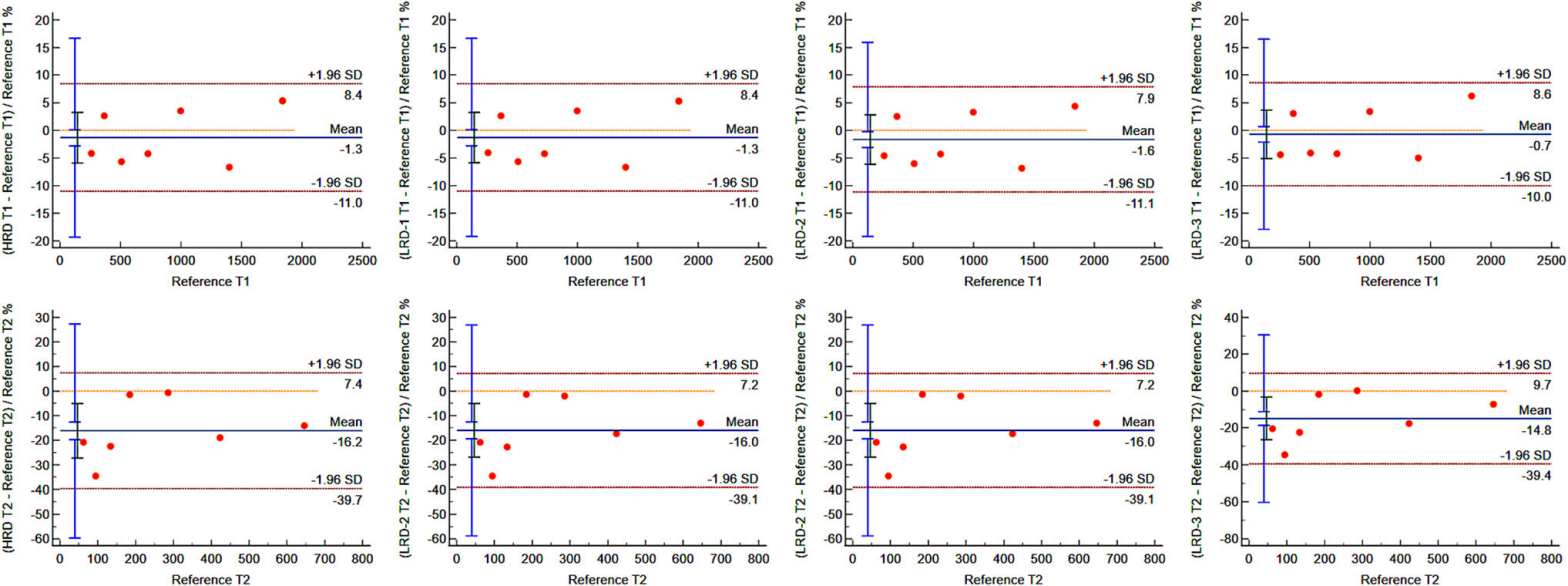
MRF accuracy from four dictionaries with the same ranges (HRD, LRD-1, LRD-2 and LRD-3) on T_1_ arrays (left side) and T_2_ arrays measurements (right side), as represented by Bland-Altman plots. Comparable relative mean differences and limits of agreements are shown in both T_1_ and T_2_ maps

The comparable linearity across dictionaries was supported by BA plots (Fig. 3) with a T_1_ array relative mean difference of −1.3% (HRD), −1.3% (LRD-1), −1.6% (LRD-2), and −0.7% (LRD-3). Meanwhile, the relative mean differences for T_2_ array were −16.2%, −16.2%, −16.0%, and −14.8%, for HRD, LRD-1, LRD-2, and LRD-3, respectively. Relaxation values of all measured arrays were within 10% [T_1_] and 24% [T_2_] limits of agreement.

**Fig. 4:**
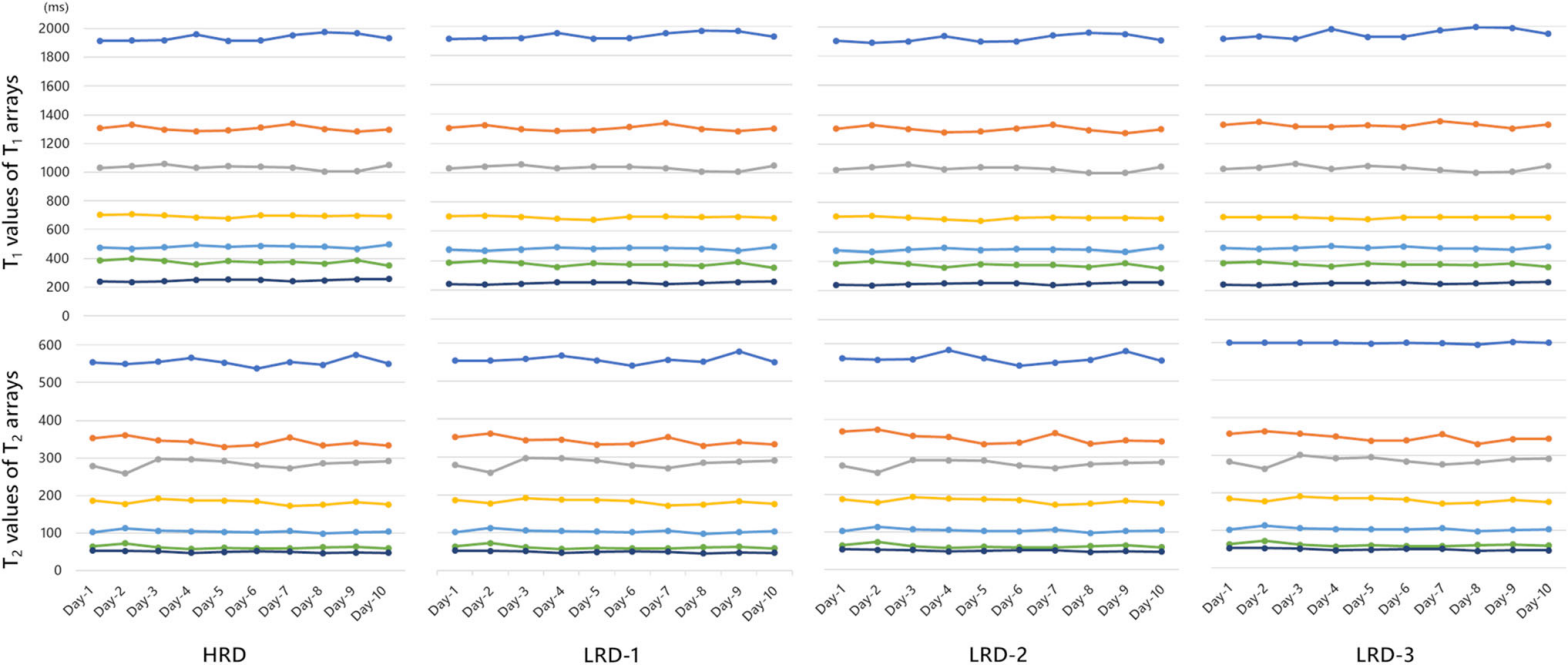
Measured T_1_ and T_2_ values of T_1_ and T_2_ phantom arrays over 10 days (average measurements from two scanners). Similar repeatabilities were apparent across four dictionaries with the same ranges (HRD, LRD-1, LRD-2, LRD-3). Mean temperature fluctuation of the two scanners was 4%

The repeatability of MRF maps for each dictionary was similar, as represented by CVs over 10 days: T_1_ CV was 2.3%, 2.3%, 2.4%, and 2.1%, and T_2_ CV was 4.3%, 4.3%, 4.6%, and 4.1%, for HRD, LRD-1, LRD-2, and LRD-3, respectively. Mean T_1_ and T_2_ measurements of phantom arrays from two scanners over 10 days are depicted in Fig. 4. During 10 days of scanning, mean (± standard deviation [SD]) phantom temperature was 23.5 ± 1.3°C on scanner A and 24.2 ± 0.6°C on scanner B.

We compared phantom measurements between the two scanners to obtain reproducibility metrics. MRF reconstructed using each dictionary yielded the comparable ICC > 0.99 for T_1_ and T_2_ measurements. T_1_ CV was 2.1%, 2.1%, 2.2%, and 2.0%, and T_2_ CV was 3.7%, 3.8%, 3.9%, and 3.6% for HRD, LRD-1, LRD-2 and LRD-3, respectively.

Dictionary with a much wider range and finer step-sizes (VHRD) demonstrated similar accuracy and interscanner reproducibility, yet seemingly higher 10 days CV of T_2_ measurements (5.3%), as shown in Supplementary Table 1. The MRF repeatability and reproducibility in the boundary slices ROIs were lower than those of phantom arrays located in a more central slice, with the 10-day CV of 3.3–4.1% (T_1_) and 4.9–8.1% (T_2_) and interscanner CV of 4.3–4.9% (T_1_) and 8.0–10.7% (T_2_). These findings were described in more detail in the Supplementary Appendix.

### Volunteer study

**Fig. 5:**
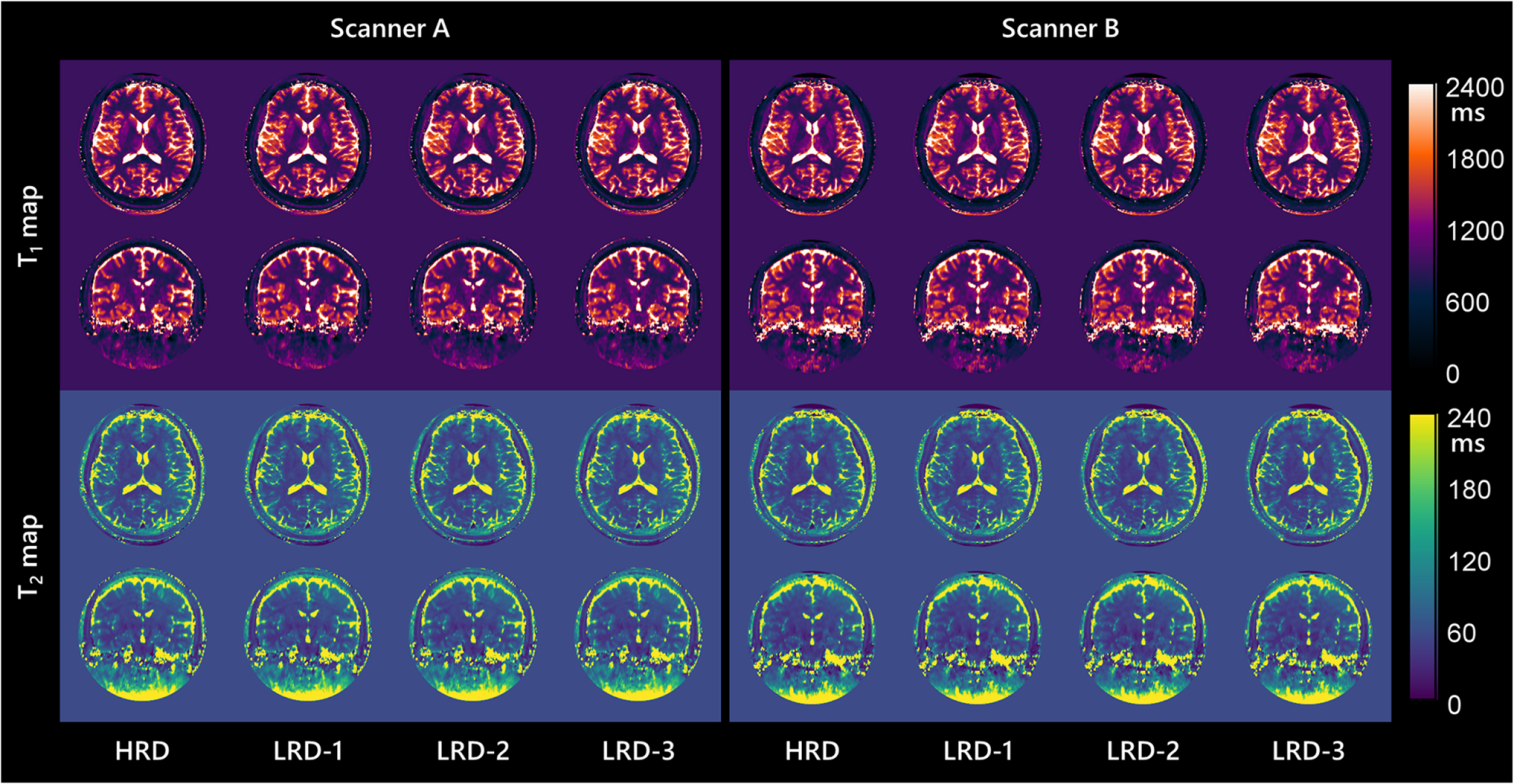
Representative full-resolution T_1_ and T_2_ maps in the native space from one healthy participant depict good details with similar consistencies between the two MR scanners (scanner A, left side; scanner B, right side) and four dictionaries with the same ranges (HRD, LRD-1, LRD-2, LRD-3)

All 39 volunteers (19 men, 20 women; mean age, 26.2 ± 4.1 years) were included in the final analysis. MRF template matching for a whole-brain scan requires around 2.4 h, 32 min, 12.6 min, and 6.3 min for HRD, LRD-1, LRD-2, and LRD-3. Meanwhile, a much broader and finer dictionary, VHRD, requires almost 59 h for template matching. Aside from template matching, the whole reconstruction process for each dictionary requires additional 3.1 h for raw data loading and processing. MRF T_1_ and T_2_ maps of one representative subject reconstructed using each dictionary are shown in Fig. 5. Average normalized MRF maps from all dictionaries demonstrated consistencies in most brain parenchymas (Supplementary Figure 1). Mean T_1_ and T_2_ values of each VOI are shown in Table 1. There is no significant difference between the four dictionaries measurements in all VOIs (ANOVA, *p* > 0.05).

**Table 1:** Mean values of T_1_ and T_2_ of each brain VOI (in milliseconds) across dictionaries with the same ranges (HRD, LRD-1, LRD-2, LRD-3). There is no significant difference between the four dictionaries’ measurements in all VOIs

		Dictionary							
Volume of interest		HRD		LRD-1		LRD-2		LRD-3	
		Mean	SD	Mean	SD	Mean	SD	Mean	SD
Cerebral white matter	T_1_	947.9	29.6	947.1	29.4	936.7	28.3	937.2	28.3
	T_2_	59.5	4.6	59.7	4.7	58.6	4.4	58.7	4.4
Cerebral grey matter	T_1_	1507.9	46.7	1505.6	46.1	1499.3	42.8	1499.9	43.1
	T_2_	101.9	8.2	101.6	8.2	99.0	7.3	99.2	7.3
Cerebellar white matter	T_1_	1070.5	38.7	1069.0	38.0	1059.4	38.2	1059.7	38.1
	T_2_	58.5	7.9	58.2	7.7	57.0	7.4	57.1	7.4
Cerebellar grey matter	T_1_	1527.0	65.4	1520.5	63.4	1518.6	61.9	1518.7	62.3
	T_2_	114.4	16.3	113.6	15.5	111.7	14.7	111.9	14.8
Thalamus	T_1_	1219.1	90.4	1211.3	87.1	1198.8	84.7	1198.8	84.7
	T_2_	61.9	6.4	63.3	6.8	59.3	6.0	59.5	6.0
Caudate nucleus	T_1_	1412.5	140.5	1402.1	128.6	1386.1	122.7	1385.8	123.1
	T_2_	86.4	15.0	88.9	17.6	82.8	9.5	83.0	9.6
Putamen	T_1_	1136.5	57.4	1136.6	57.4	1139.5	58.4	1138.3	57.6
	T_2_	52.1	4.7	52.1	4.7	52.2	4.7	52.3	4.6
Globus pallidus	T_1_	928.3	72.6	928.2	72.6	927.8	73.2	928.4	73.0
	T_2_	39.4	5.7	39.4	5.7	39.4	5.8	39.5	5.8
Hippocampus	T_1_	1486.8	70.5	1486.4	69.6	1486.8	70.0	1488.4	70.2
	T_2_	76.9	13.1	76.6	10.9	75.6	10.5	75.8	10.5
Lateral ventricle	T_1_	3726.6	269.0	3726.5	268.9	3727.2	268.8	3732.7	268.5
	T_2_	1238.1	265.1	1238.8	265.1	1239.3	264.9	1246.6	264.9

Interscanner reproducibility metrics for T_1_ and T_2_ measurements from all dictionaries were equal, as shown in Table 2. In BA analyses of brain parenchyma VOIs, relative mean differences were around 1.3%, with 11.1–11.2% limits of agreement (T_1_) and 0.3% with 19.4% limits of agreement (T_2_), as shown in Supplementary Figure 2a. ICCs were almost uniform at 0.953 (T_1_) and 0.954 (T_2_) for all dictionaries, except LRD-3 (T_2_, 0.953). The average within-subject coefficient of variation (wCV) was comparable across four dictionaries, around 1.6% (T_1_), 4.6% (T_2_) for white matter (WM) and 3.4% (T_1_), 5.5% (T_2_) for grey matter (GM).

**Table 2:** Interscanner reproducibility of 3D MRF in the human brain, evaluated using Bland-Altman plots, ICCs, and wCV across dictionaries with the same ranges (HRD, LRD-1, LRD-2, LRD-3). Despite the number of entries of each dictionary being substantially different, all dictionaries demonstrated comparable metrics, both in the brain parenchyma and CSF VOIs

		Dictionary			
		HRD	LRD-1	LRD-2	LRD-3
Brain parenchyma	**Bland-Altman**				
	**Mean difference (%)**				
	T_1_	1.3%	1.3%	1.3%	1.3%
	T_2_	0.3%	0.3%	0.3%	0.3%
	**Limits of agreement (%)**				
	T_1_	11.1%	11.2%	11.2%	11.2%
	T_2_	19.4%	19.4%	19.4%	19.4%
	**ICC**				
	T_1_	0.953	0.953	0.953	0.953
	T_2_	0.954	0.954	0.954	0.953
	**wCV (%)**				
	T_1_ WM	1.6%	1.6%	1.6%	1.6%
	T_1_ GM	3.4%	3.4%	3.4%	3.4%
	T_2_ WM	4.6%	4.6%	4.6%	4.6%
	T_2_ GM	5.5%	5.5%	5.5%	5.5%
CSF	**Bland-Altman**				
	**Mean difference (%)**				
	T_1_	1.1%	1.1%	1.1%	1.1%
	T_2_	–0.0%	–0.0%	–0.0%	–0.0%
	**Limits of agreement (%)**				
	T_1_	9.8%	9.8%	9.8%	9.7%
	T_2_	26.4%	26.4%	26.4%	25.9%
	**ICC**				
	T_1_	0.650	0.650	0.649	0.653
	T_2_	0.797	0.797	0.797	0.801
	**wCV (%)**				
	T_1_	2.5%	2.5%	2.5%	2.5%
	T_2_	7.7%	7.7%	7.7%	7.6%

No notable difference was found across dictionaries for cerebrospinal fluid (CSF) VOIs (Table 2). CSF T_1_ measurement had a relative mean difference of 1.1% with 9.7–9.8% limits of agreement, while T_2_ had a nearly 0% relative mean difference with 25.9–26.4% limits of agreement (Supplementary Figure 2b). ICCs were substantially lower than the brain parenchyma VOIs, but were similar across dictionaries, with 0.65 for T_1_ and 0.80 for T_2_. The average within-subject coefficient of variation (wCV) was about 2.5% for T_1_ and 7.6–7.7% for T_2_.

However, we got higher variabilities in CSF VOI using a wider range dictionary, VHRD (Supplementary Table 2). VHRD T_1_ relative mean difference was 2.0% with 17.5% limits of agreement, compared to 1.1% with 9.8% limits of agreement for HRD, and T_2_ relative mean difference was 1.6% (limits of agreement, 48.8%) compared to HRD mean bias of nearly 0% (limits of agreement, 26.4%). Seemingly better reproducibility metrics of HRD than VHRD in CSF VOI were also evident in wCV and ICC analyses. HRD wCVs were 2.5% and 7.7% with ICCs of 0.650, 0.797 while VHRD wCVs were 5.0% and 13.3% with ICCs of 0.511 and 0.525 for T_1_ and T_2_ measurements, respectively. The mean T_1_ value of CSF was markedly higher on VHRD (4273 ms) than HRD (3727 ms), with maximum T_1_ values of 7000 ms and 4500 ms, for VHRD and HRD, respectively. These maximum values were ascribed to the upper range of each dictionary. Notably, HRD truncated a certain range of T_1_ and T_2_ values in CSF VOI (Supplementary Figure 3).

## Discussion

This study investigated 3D MRF with four dictionary resolutions with the same ranges and evaluated three important quantitative metrics of imaging performance: accuracy, repeatability, and reproducibility. The phantom study showed that MRF has high accuracy for T_1_ measurement and modest accuracy for T_2_ measurement. MRF repeatability over 10 days of scanning was good. Meanwhile, reproducibility was evaluated through phantom and volunteer studies, showing good reproducibility. High interscanner reproducibility, particularly for T_1_ estimation, was also demonstrated in this study. Additionally, 3D MRF with a broader range dictionary was also investigated.

The high accuracy of MRF T_1_ estimations with lower accuracy for T_2_ estimations was evident in this ISMRM/NIST phantom study, consistent with previously reported results [10, 19], regardless of dictionary resolution. The supplementary information of the 2D MRF study by Ma et al [1] described that T_1_ and T_2_ measurement accuracies were not significantly affected by dictionary resolution. Our phantom results with 3D MRF supported the previous evaluation with 2D MRF. In terms of repeatability, comparable CVs were evident for all dictionaries in T_1_ and T_2_ measurements (T_1_, CV < 3%; T_2_, CV < 5%) compared to the 3D MRF study by Ma et al, which implemented B_1_ correction (T_1_, CV < 4%; T_2_, CV < 7%) [10]. Compared to T_1_ measurements, a lower interscanner reproducibility on T_2_ measurements was noted with all dictionaries. Such lower T_2_ reproducibility has been reported in the MRF literature using 2D-SSFP MRF (T_1_, CV 0.2% vs. T_2_, CV 0.7%) [13]. In the present phantom study, there were no marked differences in accuracy, repeatability, or reproducibility between dictionaries with differing resolutions and ranges.

In the human study, all dictionaries with the same ranges obtained comparable T_1_ and T_2_ values for all brain VOIs. Nevertheless, higher variability was observed in CSF relaxation values measurement than in brain parenchyma, consistent with the prior report [13, 20]. Lower reproducibility on CSF relaxometry might be attributed to lower SNR of CSF due to the relatively high flip angle of MRF pulse [13], CSF flow effects [20], and physiological inhomogeneity [21].

In addition, significant differences in CSF relaxometry measurements were seen between dictionaries with different ranges. We considered the upper dictionary limit a major contributing factor. Four dictionaries with the same ranges potentially underestimate CSF relaxometry by truncating the upper limit at 4500 ms, as VHRD measured CSF T_1_ values exceeding 4500 ms in nearly half of the subjects. Previous studies using various T_1_ mapping techniques have reported similar CSF T_1_ values with VHRD measurements (> 4000 ms) [22–24]. Accordingly, FISP-MRF literature with lower dictionary upper ranges (3000–4500 ms) also described lower T_1_ values for CSF [14, 25].

More importantly, to various extents, CSF relaxometry will also affect relaxometry measurements of brain parenchyma due to the partial volume of subarachnoid CSF, perivascular spaces, and subvoxel water content. Our data showed that in most brain parenchymal VOIs, measured maximum values differed significantly, with each close to the upper range of the respective dictionary. In elderly subjects, in whom perivascular spaces are more abundant than in our relatively young study participants, this difference could also affect mean T_1_ and T_2_ values. Thus, a broader range dictionary with a higher upper limit of T_1_ (> 4500 ms) might be advisable, especially for elderly subjects.

The seemingly better reproducibility shown by four dictionaries with the same ranges compared to VHRD in CSF T_1_ value measurements may be attributable to the truncation of the dictionary’s upper limit, yielding less variability in the measured relaxation times. Our MRF reproducibility might also have been affected by B_1_ variation. Nonetheless, our MRF measurements for grey and white matter were comparable to values reported in the literature using spin-echo techniques [24, 25], with interscanner CVs kept under 6%. A 3D MRF study without B_1_ correction reported interscanner CVs under 10% for T_1_ and T_2_ in solid brain compartments [26].

Despite the improved estimation performance, the computational expense of the dictionary-based method remains a significant barrier to MRF application in clinical practice, where parameter maps should be generated quickly. The dictionary matching process for a large dictionary (e.g., HRD and VHRD) might take more than 1 h in a typical workstation with GPU. For these reasons, the number of entries should be kept under control. Nevertheless, it must be carefully defined to fully cover the physiologic range of target tissue and avoid being excessively sparse, resulting in unacceptably large biases. The lowest resolution dictionary in our study required around 6 min for the template matching process with comparable accuracy, repeatability, and reproducibility.

## Limitations

Our study had several limitations. First, we did not perform any B_1_ correction measures, as this option was unavailable during subject enrollment. Nonetheless, the sequence became available later, and we conducted an additional phantom experiment with B_1_ correction. However, unlike prior studies [9, 10, 27], we experienced banding artifacts in the reconstructed MRF maps after B_1_ correction due to inaccurate B_1_ maps, which significantly affected the measured value. Also, the implemented 3D MRF sequence does not include any measures to correct flip angle error due to imperfect slab profile. Correspondingly, lower T_1_ and T_2_ measurement consistencies were evident in our phantom experiment using boundary slices, as described in the Supplementary Appendix.

Underestimation of T_2_ values was notable in all dictionaries, particularly in the two highest T_2_ phantom arrays. Such underestimation was consistently reported in previous studies utilizing the FISP-MRF sequence [10, 12]. As this underestimation persisted even after the B_1_ correction [12], other causes should be considered. Moreover, this effect is not apparent in different sequences, such as MRF based on echo-planar imaging (MRF-EPI) [28]. Kobayashi et al found that the T_2_ underestimation resulted from the diffusion weighting caused by the spoiler gradient used in the FISP-MRF [29]. Incorporating the diffusion effect of the spoiler gradient and ADC maps into the dictionary might be one potential solution. Furthermore, in the phantom and human study, interscanner reproducibility of T_2_ estimation was notably lower than T_1_. Such findings were consistently reported in prior MRF literature [12–14] and might pose a challenging task for future MRF optimization.

Second, we did not perform a scan-rescan of each subject in the same scanner. Intra-scanner repeatability thus could not be assessed. Also, as scans in different MR units were taken on slightly different days, physiological variations may have affected interscanner reproducibility as confounders. Physiological differences can affect brain morphometry even in same-day scans [30].

Last, the age range of volunteers in our study was relatively narrow and elderly participants were not included. Further studies are needed to investigate the clinical value of a broader MRF dictionary range, particularly in scans of elderly individuals, in whom perivascular space and subvoxel water content are more prominent.

### Conclusion

In conclusion, our study demonstrated that 3D MRF offered good accuracy, repeatability, and reproducibility, particularly for T_1_ value estimation, with comparable performance across different dictionary resolutions. A dictionary with a lower resolution but a well-defined range may be adequate, resulting in significant reductions in computational load.

## Supplementary Information

ESM 1 (DOCX 7908 kb)

## Abbreviations

2D MRF: Two-dimensional magnetic resonance fingerprinting
3D MRF: Three-dimensional magnetic resonance fingerprinting
3D-MPRAGE: Three-dimensional magnetization-prepared rapid acquisition of gradient echo
BA: Bland-Altman
CV: Coefficient of variation
DARTEL: Diffeomorphic anatomical registration through exponentiated lie algebra
EPI: Echo-planar imaging
FISP: Fast imaging with steady-state precession
GRAPPA: Generalized autocalibrating partial parallel acquisition
HRD: High-resolution dictionary
ICC: Intraclass correlation coefficient
LRD: Low-resolution dictionary
VHRD: Very high-resolution dictionary
wCV: within-subject coefficient of variation

## Declarations

### Guarantor

The scientific guarantor of this publication is Yuji Nakamoto.

### Statistics and biometry

No complex statistical methods were necessary for this paper.

### Methodology

• prospective

• cross-sectional study

• performed at one institution
