# Supplementary material for: Accuracy, repeatability, and reproducibility of T1 and T2 relaxation times measurement by 3D magnetic resonance fingerprinting with different dictionary resolutions
Source: Eur Radiol. 2022 Nov 24;33(4):2895–904. doi: 10.1007/s00330-022-09244-x (PMC10017611; doi:10.1007/s00330-022-09244-x)
Supplement: Supplementary file 1 — (DOCX 7908 kb) [file 330_2022_9244_MOESM1_ESM.docx]

**Supplementary Figure 1.**


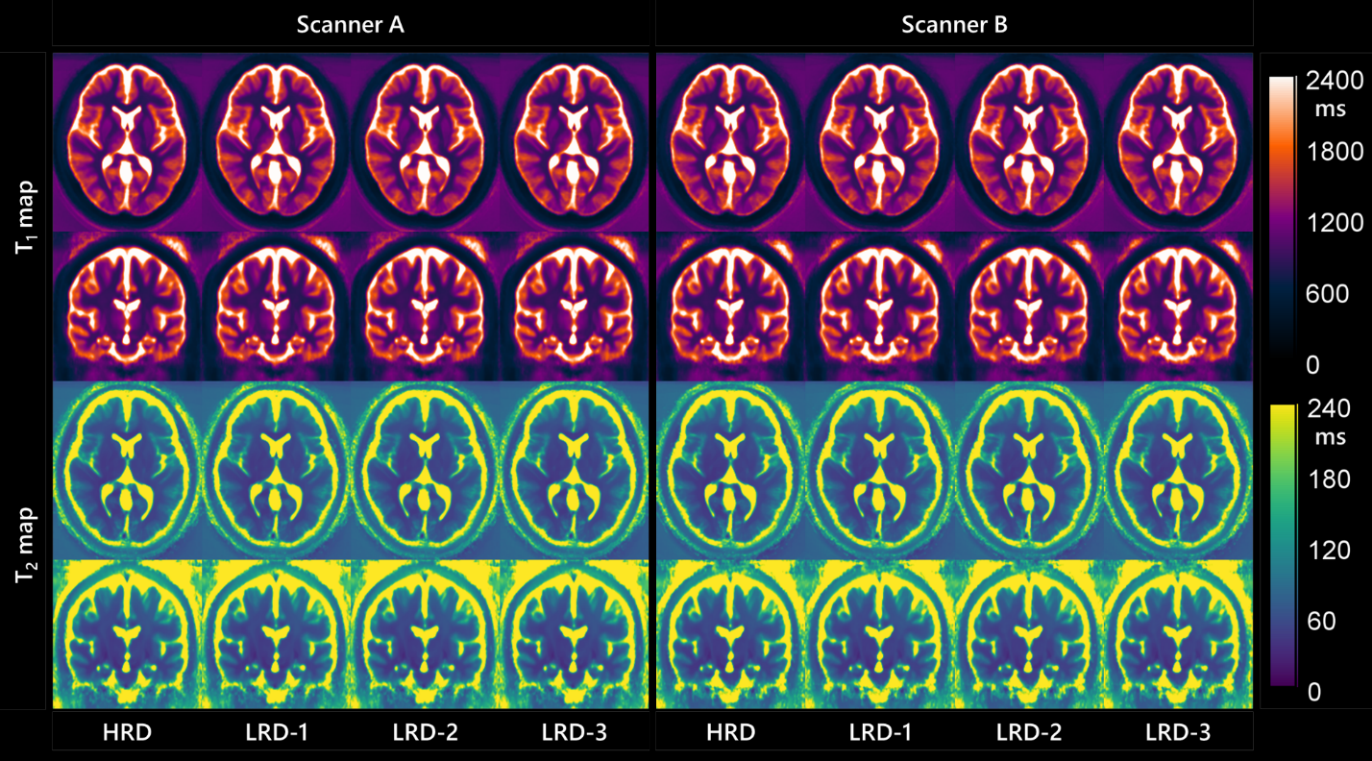


**Supplementary Figure 1.** Average spatially normalized T_1_ and T_2_ maps for all healthy volunteers reconstructed using four different dictionaries with the same ranges (HRD, LRD-1, LRD-2, LRD-3). Consistencies are evident across dictionaries.

**Supplementary Figure 2a.**


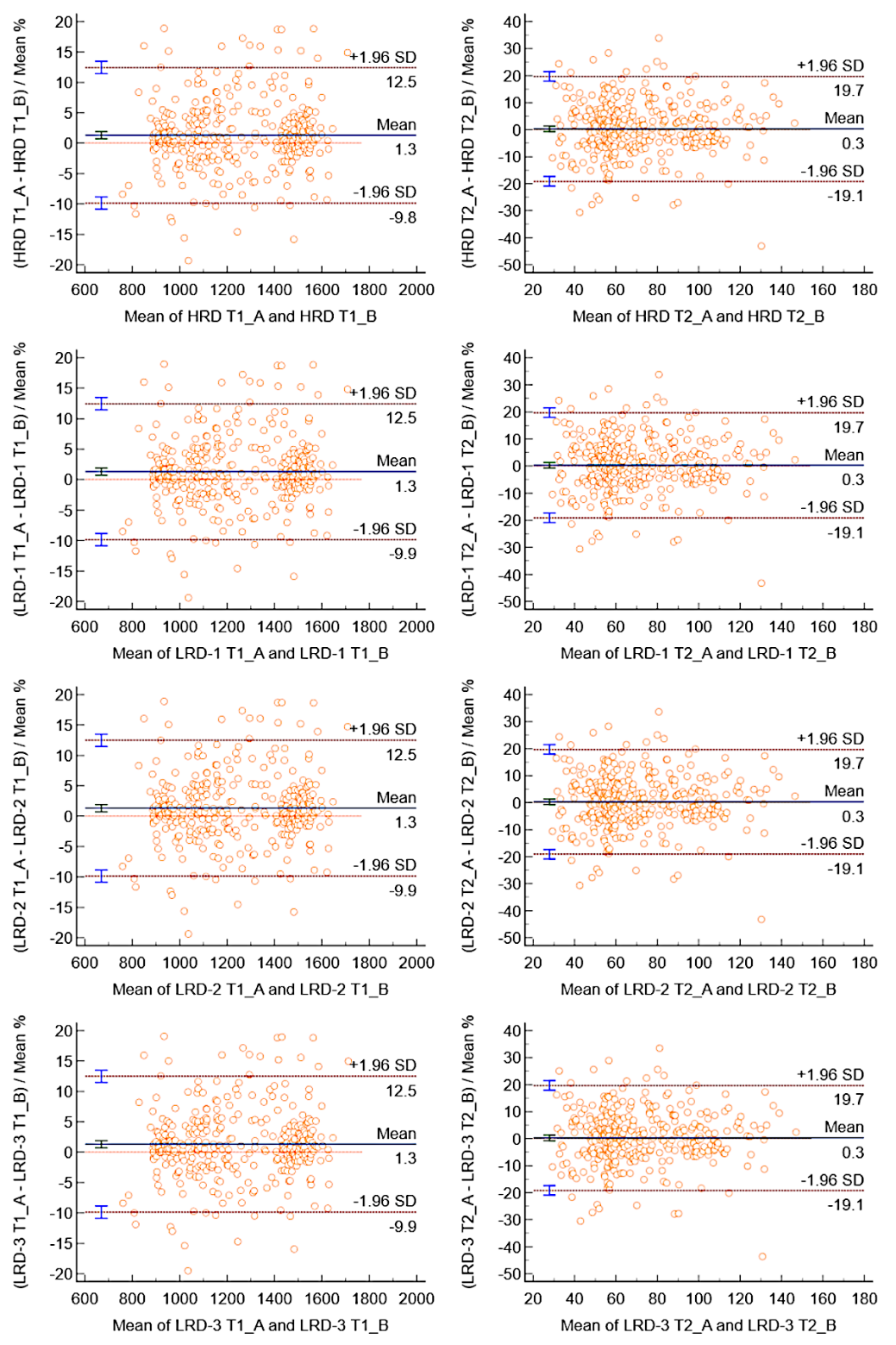


**Supplementary Figure 2a.** MRF interscanner reproducibilities on brain parenchyma VOIs were evaluated using Bland-Altman plots. Each plot compared the measured relaxational values at MR scanners A and B using one particular dictionary. Plots in the left column are for T_1_ values, and plots in the right column are for T_2_ values. Plots in the first row are from the high-resolution dictionary (HRD), and the next lower rows are low-resolution dictionary 1 (LRD-1), LRD-2 and LRD-3, respectively. Interscanner reproducibilities across dictionaries were comparable. Interscanner reproducibilities of T_1_ measurements were better than T_2_, as seen by smaller limits of agreement (around 11% vs. 19% for T_1_ and T_2_, respectively).

**Supplementary Figure 2b.**


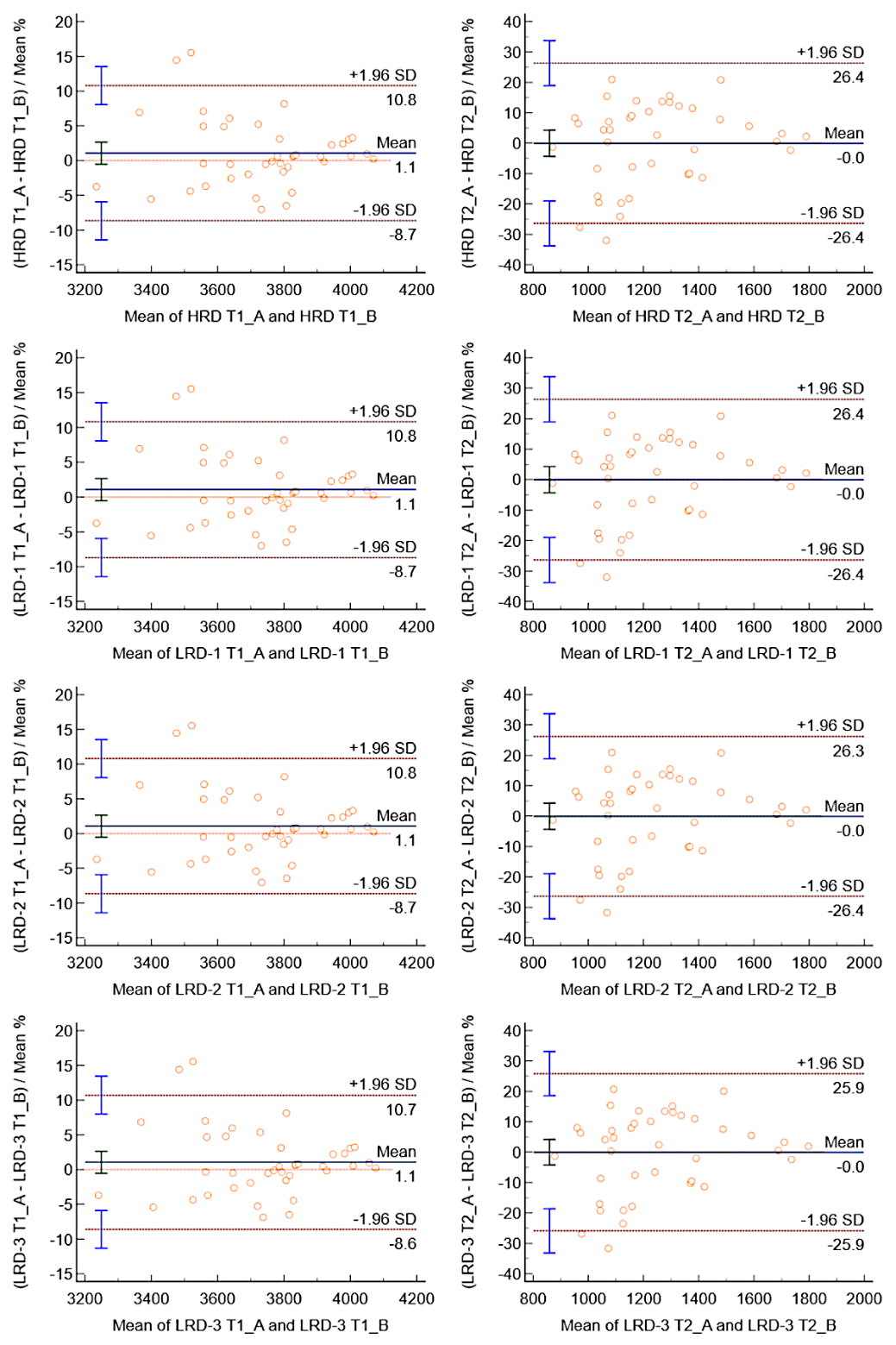


**Supplementary Figure 2b.** MRF interscanner reproducibilities on brain CSF VOIs were evaluated using Bland-Altman plots. Each plot compared the measured relaxational values at MR scanners A and B using one particular dictionary. Plots in the left column are for T_1_ values, and plots in the right column are for T_2_ values. Plots in the first row are from the high-resolution dictionary (HRD), and the next lower rows are low-resolution dictionary 1 (LRD-1), LRD-2 and LRD-3, respectively. All dictionaries showed comparable reproducibilities, with better reproducibilities on T_1_ than T_2_ measurement, similar to the results of brain parenchyma VOIs. When comparing T_2_ reproducibility to brain parenchyma VOIs, larger variabilities were observed (around 26% limits of agreement).

**Supplementary Figure 3.**


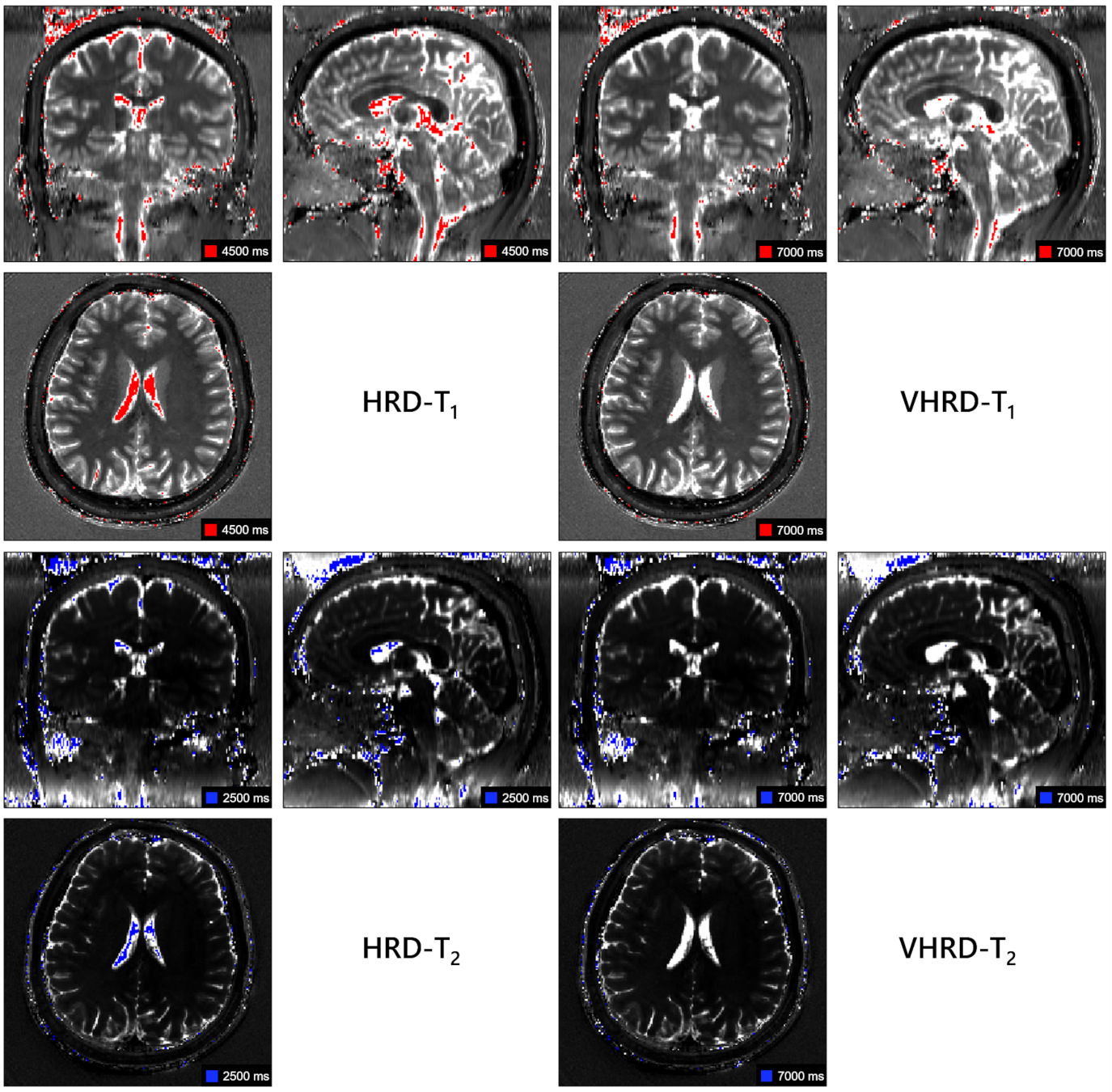


**Supplementary Figure 3.** Representative MRF T_1_ maps (upper half row) and T_2_ maps (lower half row) of one subject reconstructed using two dictionaries with different ranges (HRD, left side; VHRD, right side). Voxels with maximum values from each dictionary (HRD, T_1_ of 4500 ms and T_2_ of 2500 ms; VHRD, T_1_ of 7000 ms and T_2_ of 7000 ms) are shown in red (T_1_ maps) and blue (T_2_ maps). With HRD, many surface CSF and ventricular CSF voxels show red color on the T_1_ map and blue color on the T_2_ map. On the other hand, just a few voxels are colored red on the T_1_ map or blue on the T_2_ map with VHRD.

**Supplementary Figure 4.**


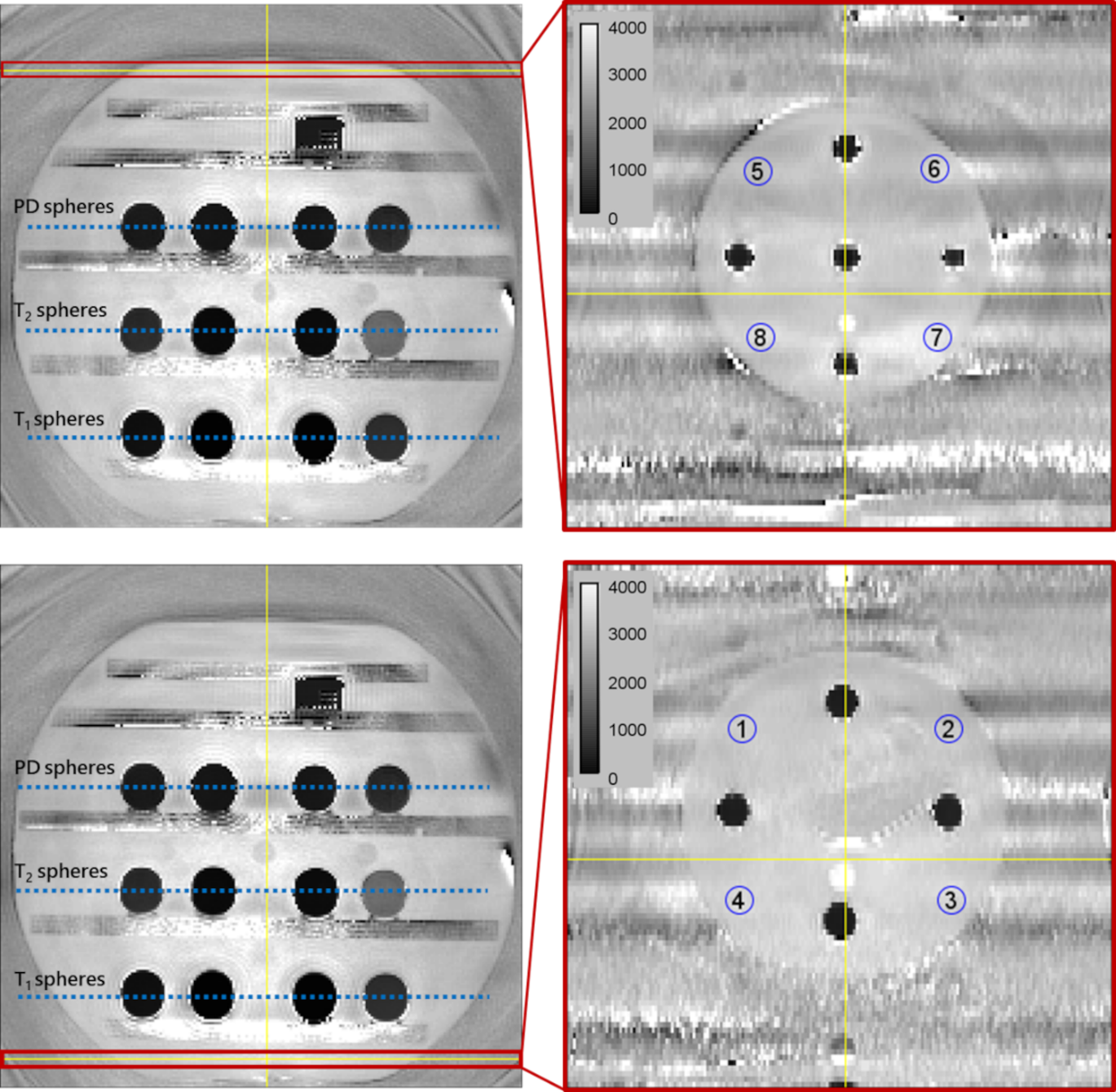


**Supplementary Figure 4.** In addition to phantom sphere ROIs located in relatively central slices (blue dotted line), we evaluated the repeatability and reproducibility of 3D MRF using 8 ROIs (blue circles) situated in the upper boundary (top row) and lower boundary (bottom row) slices, as shown by the red boxes in the T_1_ maps above.

**Supplementary Table 1.**

|  | **Dictionary** | | | | |
| --- | --- | --- | --- | --- | --- |
|  |  | | | | |
|  | VHRD | HRD | LRD-1 | LRD-2 | LRD-3 |
| **Linearity (R^2^)** |  |  |  |  |  |
| T_1_ | 0.991 | 0.992 | 0.992 | 0.992 | 0.993 |
| T_2_ | 0.985 | 0.987 | 0.987 | 0.989 | 0.988 |
| **Bland-Altman** |  |  |  |  |  |
| **Mean difference (%)** |  |  |  |  |  |
| T_1_ | -1.3% | -1.3% | -1.3% | -1.6% | -0.7% |
| T_2_ | -16.3% | -16.2% | -16.0% | -16.0% | -14.8% |
| **Limits of agreement (%)** |  |  |  |  |  |
| T_1_ | 9.7% | 9.7% | 9.7% | 9.5% | 9.3% |
| T_2_ | 23.6% | 23.6% | 23.2% | 23.2% | 24.5% |
| **Repeatability (CV)** |  |  |  |  |  |
| T_1_ | 2.7% | 2.3% | 2.3% | 2.4% | 2.1% |
| T_2_ | 5.3% | 4.3% | 4.3% | 4.6% | 4.1% |
| **Interscanner Reproducibility** |  |  |  |  |  |
| **CV (%)** |  |  |  |  |  |
| T_1_ | 2.1% | 2.1% | 2.1% | 2.2% | 2.0% |
| T_2_ | 4.0% | 3.7% | 3.8% | 3.9% | 3.6% |
| **ICC** |  |  |  |  |  |
| T_1_ | 0.999 | 0.999 | 0.999 | 0.999 | 0.999 |
| T_2_ | 0.997 | 0.998 | 0.998 | 0.998 | 0.999 |

**Supplementary Table 1.** Comparison of MRF accuracy, repeatability and interscanner reproducibility metrics on ISMRM/NIST phantom among four dictionaries with the same ranges (HRD, LRD-1, LRD-2, LRD-3) and one dictionary with a broader range (VHRD). All dictionaries demonstrated similar accuracy, repeatability and interscanner reproducibility, yet VHRD shows a seemingly higher ten-day CV of T_2_ measurements.

**Supplementary Table 2.**

|  |  |  | **Dictionary** | |
| --- | --- | --- | --- | --- |
|  |  |  | HRD | VHRD |
|  | **Brain parenchyma** | **Bland-Altman**  **Mean difference (%)** |  |  |
|  |  | T_1_ | 1.3% | 1.4% |
|  |  | T_2_ | 0.3% | 0.9% |
|  |  | **Limits of agreement (%)** |  |  |
|  |  | T_1_ | 11.1% | 11.5% |
|  |  | T_2_ | 19.4% | 21.8% |
|  |  | **ICC** |  |  |
|  |  | T_1_ | 0.953 | 0.949 |
|  |  | T_2_ | 0.954 | 0.910 |
|  |  | **wCV (%)** |  |  |
|  |  | T_1_ WM | 1.6% | 1.6% |
|  |  | T_1_ GM | 3.4% | 3.5% |
|  |  | T_2_ WM | 4.6% | 5.0% |
|  |  | T_2_ GM | 5.5% | 5.7% |
|  | **CSF** | **Bland-Altman**  **Mean difference (%)** |  |  |
|  |  | T_1_ | 1.1% | 2.0% |
|  |  | T_2_ | -0.0% | 1.6% |
|  |  | **StdDev difference (%)** |  |  |
|  |  | T_1_ | 9.8% | 17.5% |
|  |  | T_2_ | 26.4% | 48.8% |
|  |  | **ICC** |  |  |
|  |  | T_1_ | 0.650 | 0.511 |
|  |  | T_2_ | 0.797 | 0.525 |
|  |  | **wCV (%)** |  |  |
|  |  | T_1_ | 2.5% | 5.0% |
|  |  | T_2_ | 7.7% | 13.3% |

**Supplementary Table 2.** Interscanner reproducibility of 3D MRF in the human brain, evaluated using Bland-Altman plots, ICCs and wCV across dictionaries with different upper ranges (HRD, T_1_ 4500 ms, T_2_ 2500 ms; VHRD, T_1_ 7000 ms, T_2_ 7000 ms). Despite both dictionaries having comparable reproducibility in the brain parenchyma, HRD showed seemingly better reproducibility metrics in CSF VOI.

**Supplementary Table 3**

| **ROI** | **T_1_  values** | | | | **T_2_  values** | | | |
| --- | --- | --- | --- | --- | --- | --- | --- | --- |
|  | **HRD** | **LRD-1** | **LRD-2** | **LRD-3** | **HRD** | **LRD-1** | **LRD-2** | **LRD-3** |
| **ROI-1** | 3161 ± 167 | 3162 ± 166 | 3168 ± 172 | 3188 ± 193 | 2336 ± 170 | 2347 ± 155 | 2294 ± 167 | 2258 ± 234 |
| **ROI-2** | 3171 ± 135 | 3170 ± 138 | 3172 ± 136 | 3177 ± 189 | 2292 ± 141 | 2296 ± 143 | 2277 ± 122 | 2253 ± 216 |
| **ROI-3** | 3357 ± 129 | 3355 ± 129 | 3367 ± 132 | 3409 ± 143 | 2052 ± 305 | 2052 ± 303 | 2014 ± 284 | 1954 ± 257 |
| **ROI-4** | 3299 ± 154 | 3301 ± 154 | 3304 ± 149 | 3359 ± 208 | 2263 ± 205 | 2274 ± 198 | 2260 ± 182 | 2158 ± 289 |
| **ROI-5** | 3169 ± 133 | 3169 ± 133 | 3170 ± 134 | 3200 ± 195 | 2348 ± 160 | 2347 ± 159 | 2252 ± 273 | 2228 ± 261 |
| **ROI-6** | 3143 ± 138 | 3144 ± 137 | 3143 ± 138 | 3176 ± 170 | 2326 ± 146 | 2329 ± 149 | 2299 ± 200 | 2242 ± 226 |
| **ROI-7** | 3376 ± 186 | 3376 ± 187 | 3380 ± 185 | 3368 ± 189 | 2397 ± 149 | 2394 ± 145 | 2335 ± 213 | 2352 ± 195 |
| **ROI-8** | 3327 ± 102 | 3326 ± 103 | 3333 ± 99 | 3397 ± 131 | 2408 ± 111 | 2407 ± 111 | 2326 ± 203 | 2291 ± 212 |

**Supplementary Table 3.** Mean and standard deviation of T_1_ and T_2_ values (in milliseconds) of 8 ROIs located in the boundary slices for 10-day measurements.

**Supplementary Appendix**

***MRF repeatability and reproducibility in the boundary slices***

In addition to seven phantom arrays with the highest T_1_ and T_2_ values, we performed additional repeatability and reproducibility evaluation using 8 ROIs in the boundary slices, as depicted in Supplementary Figure 4. Average T_1_ and T_2_ values of those ROIs in the boundary slices for 10-day measurements were presented in Supplementary Table 3. We observed lower repeatability and interscanner reproducibility for ROIs in the boundary slices compared to phantom arrays located in a more central slice.

Ten-day repeatability (CVs) of the those ROIs in the boundary slices were 3.3%, 3.3%, 3.5%, 4.1% (T_1_) and 5.0%, 5.1%, 4.9%, 8.1% (T_2_), for HRD, LRD-1, LRD-2 and LRD-3, respectively. Meanwhile, for the phantom arrays, ten-day CVs were around 2% (T_1_) and 4% (T_2_) in all dictionaries. The interscanner CVs of the those ROIs in the boundary slices were 4.3%, 4.3%, 4.3%, 4.9% (T_1_) and 8.3%, 8.0%, 9.6%, 10.7% (T_2_), for HRD, LRD-1, LRD-2 and LRD-3, respectively. In comparison, interscanner CVs were kept at less than 3% (T_1_) and 4% (T_2_) in phantom array ROIs. The lowest resolution dictionary (LRD-3) showed a markedly higher ten-day CV and interscanner CV for T_2_ measurements in boundary ROIs. Since the reference values for such regions outside the spheres are not provided by the phantom manufacturer, we could not assess the MRF accuracy in the boundary slices.

As described in the literature [10, 27], imperfect slab profile and under-flip of the RF excitation in the boundary slice could impair T_1_ and T_2_ estimation in the boundary slice of the MRF data, leading to lower measurement accuracy and consistency. A prior study corrected the flip angle error using a 3D slab-selective RF pulse with an optimized excitation profile and premeasured 3D B_1_ map, of which B_1_ values were then simulated into the dictionary [10].
